# Nomogram for predicting occurrence of synchronous liver metastasis in colorectal cancer: a single-center retrospective study based on pathological factors

**DOI:** 10.1186/s12957-022-02516-2

**Published:** 2022-02-19

**Authors:** Yunxiao Liu, Yuliuming Wang, Hao Zhang, Mingyu Zheng, Chunlin Wang, Zhiqiao Hu, Yang Wang, Huan Xiong, Hanqing Hu, Qingchao Tang, Guiyu Wang

**Affiliations:** Department of Colorectal Surgery, the Second Affiliated Hospital of Harbin Medical University, Harbin, China

**Keywords:** Colorectal cancer, Synchronous liver metastasis, Pathological factors, Nomogram

## Abstract

**Purpose:**

The purpose of this study was to explore the risk factors for synchronous liver metastasis (LM) of colorectal cancer (CRC) and to construct a nomogram for predicting the occurrence of synchronous LM based on baseline and pathological information.

**Methods:**

The baseline and pathological information of 3190 CRC patients were enrolled in the study from the Department of Colorectal Surgery, the Second Affiliated Hospital of Harbin Medical University between 2012 and 2020. All patients were divided into development and validation cohorts with the 1:1 ratio. The characters of LM and none-LM patients in newly diagnosed colorectal cancer were utilized to explore the risk factors for synchronous LM with the univariate and multivariate logistic regression analyses. A predictive nomogram was constructed by using an R tool. In addition, receiver operating characteristic (ROC) curves was calculated to describe the discriminability of the nomogram. A calibration curve was plotted to compare the predicted and observed results of the nomogram. Decision-making curve analysis (DCA) was used to evaluate the clinical effect of nomogram.

**Results:**

The nomogram consisted of six features including tumor site, vascular invasion (VI), T stage, N stage, preoperative CEA, and CA-199 level. ROC curves for the LM nomogram indicated good discrimination in the development (AUC = 0.885, 95% CI 0.854–0.916) and validation cohort (AUC = 0.857, 95% CI 0.821–0.893). The calibration curve showed that the prediction results of the nomogram were in good agreement with the actual observation results. Moreover, the DCA curves determined the clinical application value of predictive nomogram.

**Conclusions:**

The pathologic-based nomogram could help clinicians to predict the occurrence of synchronous LM in postoperative CRC patients and provide a reference to perform appropriate metastatic screening plans and rational therapeutic options for the special population.

## Introduction

Colorectal cancer (CRC) is the third most common malignancy in the world, with high incidence and mortality globally. Metastases is one of the most common contributors for death in CRC, of which liver metastasis (LM) is the most fatal [1]. At present, research on LM in CRC patients is continuing [2–4]. It is important to note that LM are found in more than 25% of patients with CRC at the time of their first diagnosis and occur in up to 25% of patients following removal of the primary tumor [5, 6]. The 5-year survival rate for patients with CRC is approximately 56%, but can be significantly reduced when distant metastases are detected [7, 8].

LM of CRC can be divided into synchronous and metachronous LM [9]. Synchronous LM was defined as LM detected at the time of diagnosis of the primary tumor or within 6 months after diagnosis. *Jennie et al* found that up to 18.3% of patients developed LM after radical CRC surgery. Therefore, early detection of high-risk postoperative populations for synchronous LM can help physicians to improve survival by targeting screening and individualizing treatment. With the development of scientific research capacity, more and more risk factors affecting LM of CRC have been discovered such as T stage, N stage, tumor site, preoperative CEA level, and so on [10, 11]. However, most studies did not include vascular invasion (VI) as a pathological factor. Both the Association of Directors of Anatomic and Surgical Pathology [12] and the College of American Pathologists [13] emphasis is placed on documenting VI during routine pathological examination of cancer specimens. These institutions emphasized that VI was an independent predictor of poor prognosis and increased risk of LM because VI necessarily increased the risk of tumor cell entry into the bloodstream. Many studies have reached similar conclusions [14, 15]. In addition, VI is an indication for adjuvant chemotherapy in stage II patients. Therefore, there are sufficient and necessary reasons to consider VI as a risk predictor for synchronous LM. Nomogram is a kind of graphical prediction model with friendly interface, which has strong clinical application value. By assigning points, we can not only observe the influence of certain parameters but also predict the probability of a particular event from the total score. To our knowledge, this is the first nomogram including VI to predict synchronous LM of CRC.

## Methods

### Patients

Three thousand one hundred ninety CRC cases were collected from the department of colorectal surgery, the Second Affiliated Hospital of Harbin Medical University between 2012 and 2020.

Inclusion criteria included (1) patients diagnosed with CRC and underwent surgery; (2) aged ≥ 18 years old; (3) Patients with complete pathological information; (4) CRC was the only primary malignancy. Exclusion criteria included (1) patients received preoperative neoadjuvant chemoradiotherapy; (2) the baseline and pathological information of the patient was incomplete; (3) distant metastases other than LM, and (4) postoperative resection margin of tumor was positive.

The colorectal cancer patients included in this study were treated with surgery only before the diagnosis of liver metastasis.

### Variables

According to our study, age was regrouped into < 60, 60–74, and ≥ 75 years old; sex was classified as male and female; BMI was recorded as < 25 and ≥ 25; tumor size was divided into two groups: ≤ 5 cm and > 5 cm. The tumor site was grouped into right-sided colon (cecum, ascending colon, hepatic flexure, and transverse colon) and left-sided colon (splenic flexure, descending colon and sigmoid colon). The histology variable was classified as “adenocarcinoma,” “mucinous adenocarcinoma,” or “others”; the grade variable was classified as I/II and III/IV (“well-differentiated/moderately differentiated,” and “poorly differentiated/undifferentiated”) stage and the tumor type variable was classified as “ulcer type,” “uplift type,” and “infiltrating type.” Similarly, T stages, N stages, and lymph nodes examined (LNE) are grouped. The most prominent variable that VI, nerve invasion, and lymphatic invasion were classified as “yes” or “no.” The preoperative CEA level variable was classified as “positive” (≥ 5 ng/ml) and “negative” (< 5 ng/ml). In the same way, CA199 level variable was classified as “positive” (≥ 37 U/ml) and “negative” (< 37 U/ml).

### Statistical analysis

All statistical analyses were performed by R tool and SPSS 22.0. In this study, all patients were randomly (1:1 ratio) divided into development and validation cohorts and summarized by number and percentage. The characters of LM and none-LM patients in newly diagnosed colorectal cancer were utilized to explore the risk factors for synchronous LM with the univariate and multivariate logistic regression analyses. Nomogram was constructed based on the results of multivariate regression model and its performance was further evaluated by calibration and ROC. The DCA curve was used to evaluate the clinical decision ability of the model. In addition, all variables were assigned and the best cutoff value was calculated based on the total score by Youden’s index. The difference was considered statistically significant for a two-sided *P* < 0.05.

## Results

### Patients’ characteristics

A total of 3190 patients were divided evenly into the development and validation cohorts. There were 104(6.5%) LM patients in development cohort and 107(6.7%) LM patients in validation cohort. In all patients, the majority of patients were men, aged 60–74 and had a BMI of less than 25. Overall, the main proportions of the patients were associated with rectum, tumor size ≤ 5 cm, ulcer type, grade I/II, adenocarcinoma, preoperative CEA and CA-199 level negative, T4 stage, N0 stage, and LNE ≥ 12. In the encroachment around, VI accounted for 28.8% and 30.5% in the development and validation cohorts. The detailed data was summarized in Table 1.

**Table 1:** Baseline characteristics of CRC patients in our study

Characteristics	Development cohort (***n***, %)	Validation cohort (***n***, %)	***P*** value
**Age (years)**			**0.923**
< 60	645 (40.4)	644 (40.4)	
60–74	737 (46.2)	745 (46.7)	
≥ 75	213 (13.4)	206 (12.9)	
**BMI**			**0.273**
< 25	1123 (70.4)	1151 (72.2)	
≥ 25	472 (29.6)	444 (27.8)	
**Sex**			**0.054**
Male	1011 (63.4)	958 (60.1)	
Female	584 (36.6)	637 (39.9)	
**Tumor site**			**0.663**
Right-sited colon	353 (22.1)	373 (23.4)	
Left-sited colon	376 (23.6)	373 (23.4)	
Rectum	866 (54.3)	849 (53.2)	
**Tumor size (cm)**			**0.187**
≤ 5	915 (57.4)	878 (55.0)	
> 5	680 (42.6)	717 (45.5)	
**Tumor type**			**0.671**
Ulcer type*	1152 (72.2)	1170 (73.4)	
Uplift type*	430 (27.0)	415 (26.0)	
Infiltrating type*	13 (0.8)	10 (0.6)	
**Grade**			**0.678**
I/II	1376 (86.3)	1384 (86.8)	
III/IV	219 (13.7)	211 (13.2)	
**Histology**			
Adenocarcinoma	1288 (80.8)	1270 (79.6)	**0.726**
Mucinous	290 (18.2)	307 (19.2)	
Other	17 (1.1)	18 (1.1)	
**T**			**0.128**
T1/T2	172 (10.8)	166 (10.4)	
T3	703 (44.1)	653 (40.9)	
T4	720 (45.1)	776 (48.7)	
**N**			**0.839**
N0	1009 (63.3)	1014 (63.6)	
N1	372 (23.3)	378 (23.7)	
N2	214 (13.4)	203 (12.7)	
**LNE**			
< 12	262 (16.4)	274 (17.2)	
≥ 12	1333 (83.6)	1321 (82.8)	
**Vascular invasion**			**0.295**
No	1135 (71.2)	1108 (69.5)	
Yes	460 (28.8)	487 (30.5)	
**Nerve invasion**			**0.667**
No	682 (42.8)	670 (42.0)	
Yes	913 (57.2)	925 (58.0)	
**Lymphatic invasion**			**0.499**
No	1071 (67.1)	1053 (66.0)	
Yes	524 (32.9)	542 (34.0)	
**CEA**			**0.472**
Positive	504 (31.6)	523 (32.8)	
Negative	1091 (68.4)	1072 (67.2)	
**CA199**			**0.516**
Positive	241 (15.1)	228 (14.3)	
Negative	1354 (84.9)	1367 (85.7)	
**Liver metastasis**			**0.831**
Yes	104 (6.5)	107 (6.7)	
No	1491 (93.5)	1488 (93.3)	

Ulcer type*: the tumor grew into the intestinal lumen. Uplift type*: the tumor is infiltrating around the intestinal wall. Infiltrating type*: tumors grow deep into the intestinal wall and invade outwards and appears to be marginal and deep at the bottom

Construction and validation of nomogram to predict LM probability

Univariate and multivariate logistic regression analyses were performed to determine the independent risk factors for LM. In univariate analysis, the candidate predictors for the model were age, BMI, sex, tumor size and site, tumor type, grade, histology, T stage, N stage, LNE, VI, nerve invasion, lymphatic invasion, preoperative CEA, and CA-199 level. All the predictors except for age, BMI, sex, tumor type, histology, and LNE were of statistical significance in the development cohort, which were then further analyzed by multivariate logistic regression model. And the results indicated that tumor site (OR = 2.228, 95%CI = 1.272–3.901 for right-sited colon, *P* = 0.005; OR = 1.635, 95%CI = 0.889–3.007 for left-sited colon, *P* = 0.114; using rectum as the reference), VI (OR = 1.965, 95%CI = 1.182–3.266 for yes, *P* = 0.009; using no as the reference), T stage (OR = 0.130, 95%CI = 0.017–1.014 for T1/T2, *P* = 0.052; OR = 0.417, 95%CI = 0.245–0.710 for T3, *P* = 0.001; using T4 as the reference), N stage (OR = 1.252, 95%CI = 0.665–2.359 for N1, *P* = 0.487; OR = 4.071, 95%CI = 2.117–7.830 for N2, *P* < 0.001; using N0 as the reference), CEA level (OR = 3.043, 95%CI = 1.836–5.043 for CEA positive, *P* < 0.001, using CEA negative as the reference), CA-199 level (OR = 6.006, 95%CI = 3.697–9.756 for CA-199 positive, *P* < 0.001, using CA-199 negative as the reference) were independent risk factors in predicting LM (Table 2).

**Table 2:** Logistic regression analysis of the risk factors for LM in CRC patients

Characteristics	Univariate analysis		Multivariate analysis	
	OR [95% CI]	***P*** value	OR [95% CI]	***P*** value
**Age (years)**				
< 60	0.966 [0.526–1.774]	0.912		
60–74	0.858 [0.469–1.572]	0.621		
≥ 75	Ref			
**BMI**				
< 25	1.151 [0.538–1.151]	0.538		
≥ 25	Ref			
**Sex**				
Male	1.004 [0.664–1.516]	0.987		
Female	Ref			
**Tumor site**				
Right-sited colon	2.787 [1.764–4.402]	**< 0.001**	2.228 [1.272–3.901]	**0.005**
Left-sited colon	1.446 [0.857–2.441]	0.168	1.635 [0.889–3.007]	0.114
Rectum	Ref		Ref	
**Tumor size (cm)**				
≤ 5	0.670 [0.450–0.998]	**0.049**	1.033 [0.638–1.671]	0.896
> 5	Ref		Ref	
**Tumor type**				
Ulcer type	0.438 [0.096–2.009]	0.288		
Uplift type	0.226 [0.047–1.102]	0.066		
Infiltrating type	Ref			
**Grade**				
I/II	Ref		Ref	
III/IV	2.793 [1.779–4.384]	**< 0.001**	0.982 [0.555–1.737]	0.949
**Histology**				
Adenocarcinoma	0.297 [0.083–1.055]	0.060		
Mucinous	0.421 [0.113–1.568]	0.197		
Others	Ref			
**T**				
T1/T2	**0.049 [0.007–0.354]**	**0.003**	0.130 [0.017–1.014]	0.052
T3	0.321 [0.203–0.507]	**< 0.001**	0.417 [0.245–0.710]	**0.001**
T4	Ref		Ref	
**N**				
N0	0.123 [0.077–0.199]	**< 0.001**	0.246 [0.128–0.472]	**< 0.001**
N1	0.263 [0.156–0.443]	**< 0.001**	0.308 [0.165–0.573]	**< 0.001**
N2	Ref		Ref	
**LNE**				
< 12	0.920 [0.531–1.595]	0.767		
≥ 12	Ref			
**Vascular invasion**				
No	0.258 [0.171–0.387]	**< 0.001**	0.509 [0.306–0.846]	**0.009**
Yes	Ref		Ref	
**Nerve invasion**				
No	0.402 [0.253–0.637]	**< 0.001**	1.050 [0.594–1.856]	0.866
Yes	Ref		Ref	
**Lymphatic invasion**				
No	0.547 [0.367–0.817]	**0.003**	1.383 [0.810–2.363]	0.235
Yes	Ref		Ref	
**CEA**				
Positive	0.148 [0.095–0.232]	**< 0.001**	0.329 [0.198–0.545]	**< 0.001**
Negative	Ref		Ref	
**CA199**				
Positive	0.080 [0.052–0.123]	**< 0.001**	0.167 [0.103–0.270]	**< 0.001**
Negative	Ref		Ref	

**Fig. 1:**
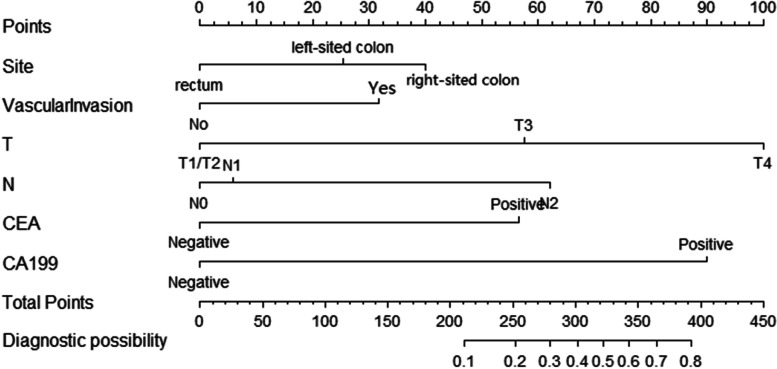
Nomogram for predicting the probability of LM

**Fig. 2:**
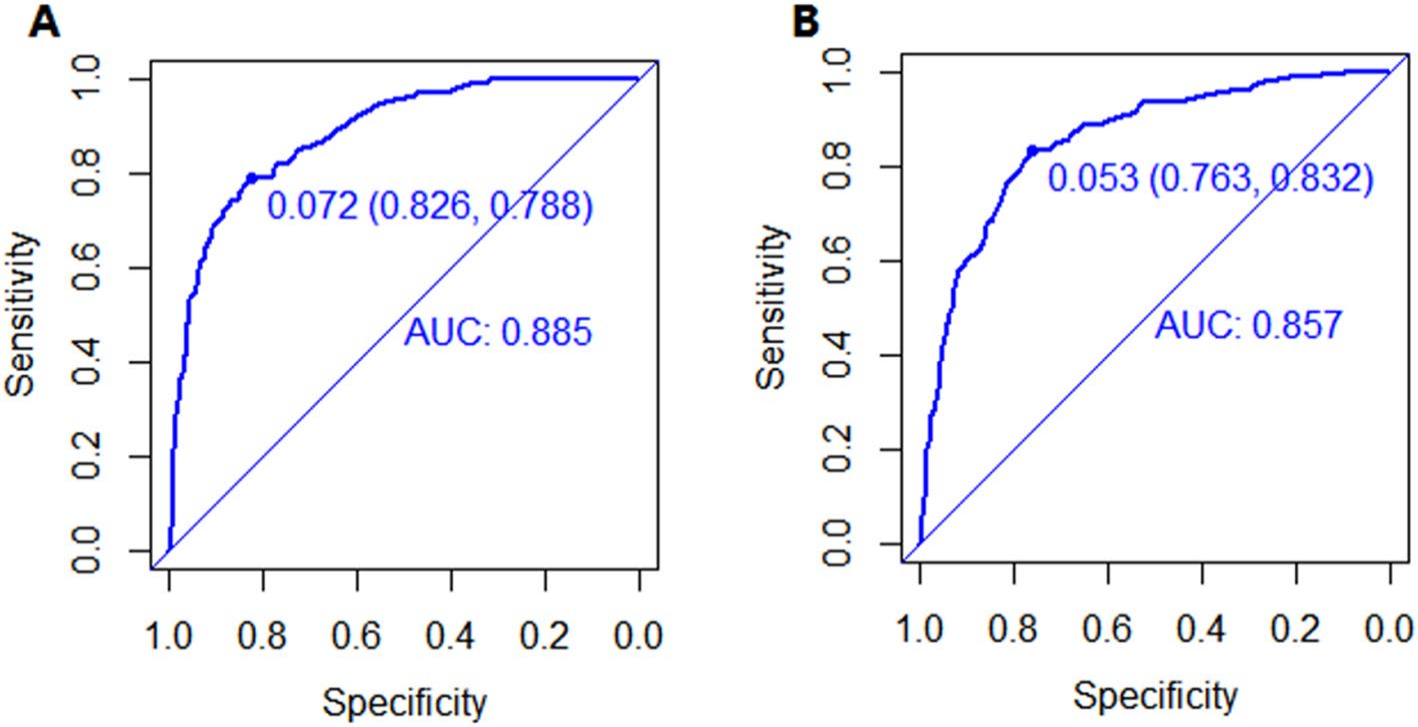
The ROC curves of nomogram for predicting LM in the development cohort (**A**) and validation cohort (**B**)

**Fig. 3:**
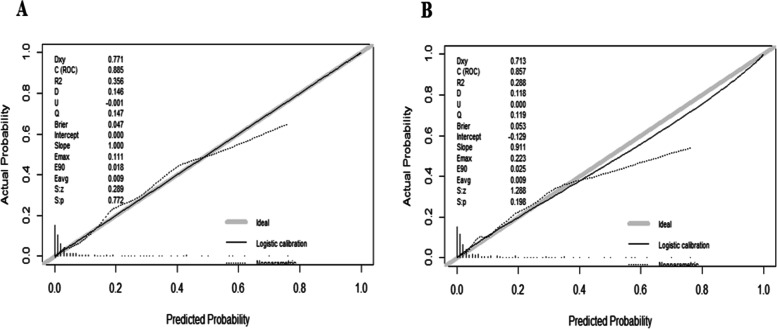
The calibration curves of the nomogram for predicting LM in the development cohort (**A**) and validation cohort (**B**)

**Fig. 4:**
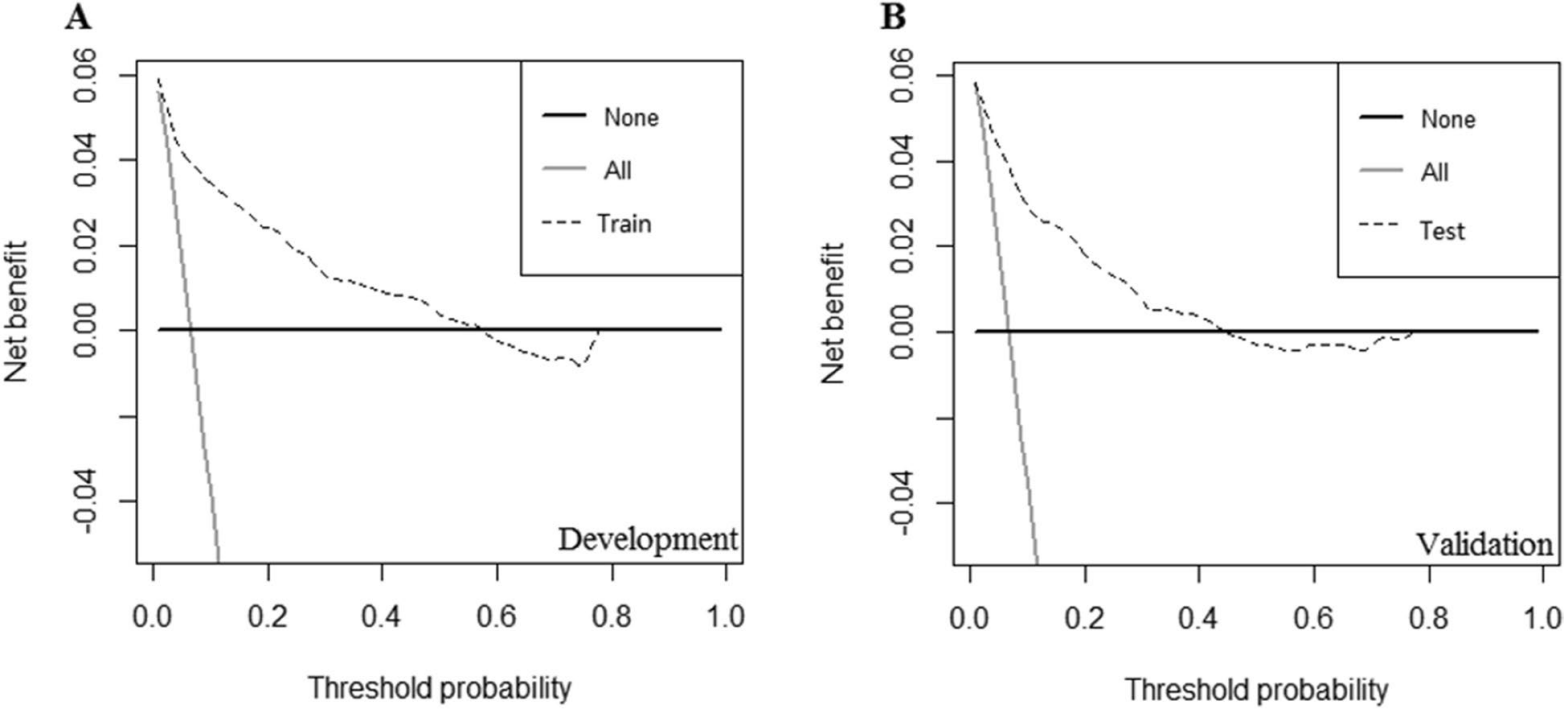
The DCA curves of the nomogram for predicting the occurrence of LM in the development cohort (**A**) and validation cohort (**B**)

Subsequently, we constructed a nomogram to predict LM for postoperative patients based on independent risk factors (tumor site, VI, T stage, N stage, CEA, and CA-199 level) (Fig. 1). The AUCs for development and validation cohort were 0.885 (95%CI = 0.854–0.916) and 0.857 (95%CI = 0.821–0.893), respectively (Fig. 2). The calibration curves (development cohort: *p* = 0.772; validation cohort: *p* = 0.198) showed the relatively satisfactory prediction accuracy of the nomogram (Fig. 3). In addition, the DCA curve also indicated good clinical practicability in both cohorts (Fig. 4).

**Fig. 5:**
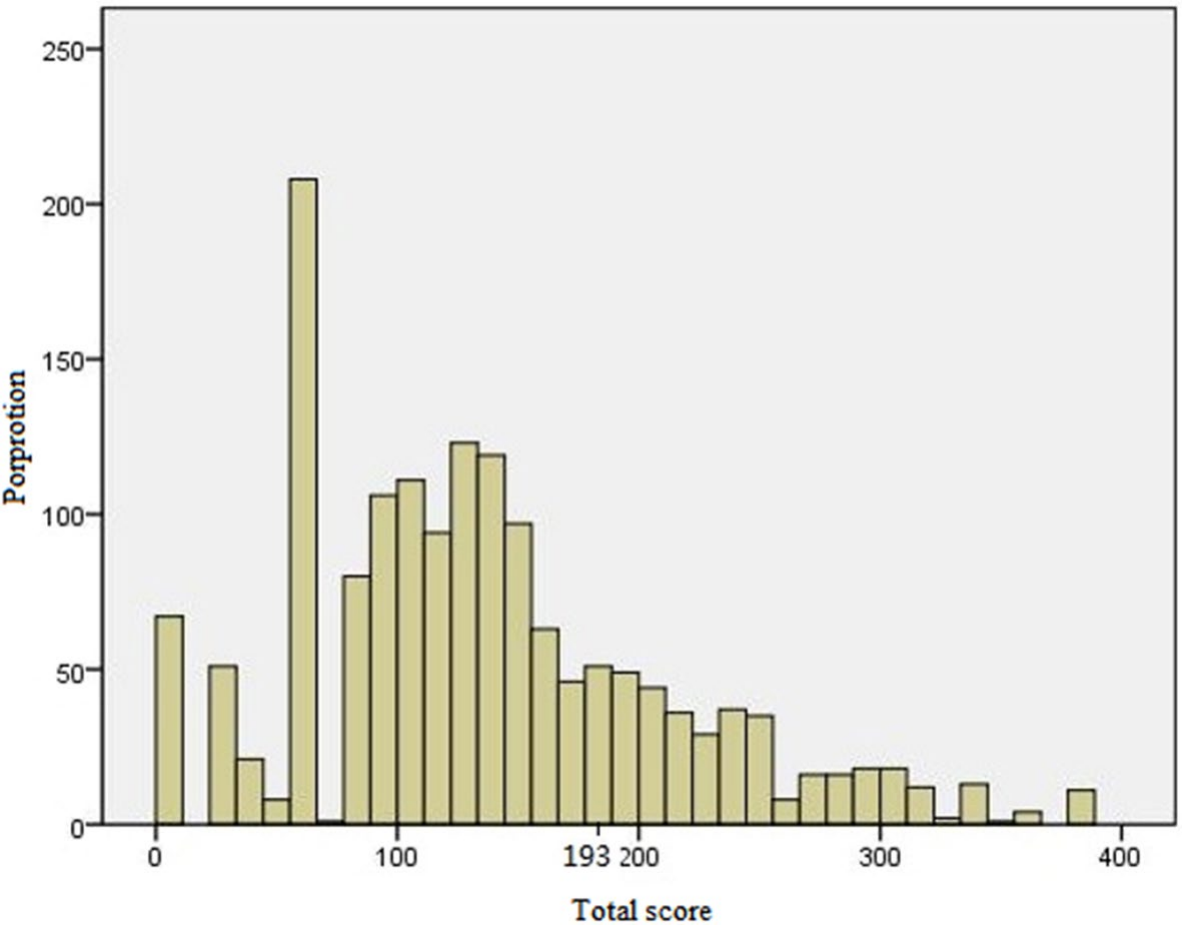
Calculate the cutoff value in LM patients

**Fig. 6:**
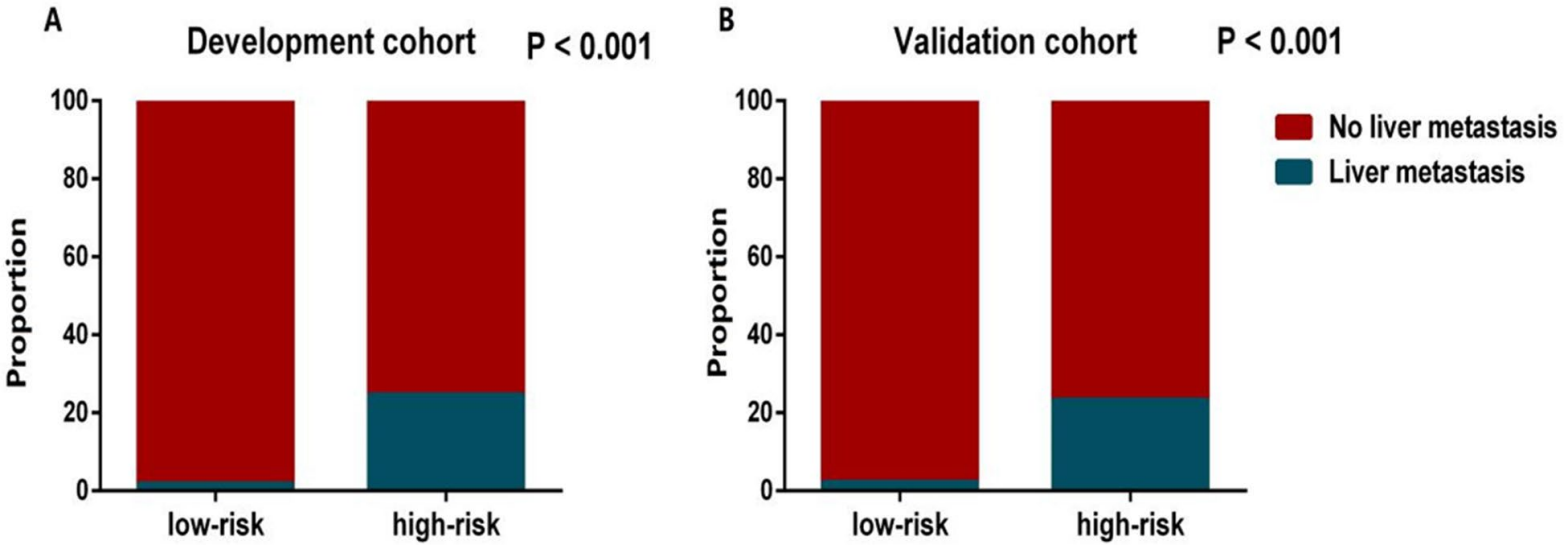
The proportion for LM patients in the low- and high-risk groups in the development cohort (**A**) and validation cohort (**B**)

Using the nomogram derived scores, all LM patients were classified into two subgroup low-risk (risk score ≤ 193) and high-risk groups (risk score > 193) (Fig. 5). And we found there were significant differences in the occurrence of LM between the high- and low-risk groups in development cohort (*P* < 0.001) and validation cohort (*P* < 0.001) (Fig. 6).

## Discussion

CRC is one of the major causes of cancer morbidity and mortality both in the worldwide. Distant metastasis disease is the main cause of poor prognosis in patients with CRC and the liver is the most common organ for metastasis. Over the course of the disease, a total of 50% of patients with CRC are likely to develop LM [16]. And the death rate remains high [17]. This also explains the necessity of this study. Nowadays, many studies have identified many independent factors for LM in patients with CRC but few studies developed predictive nomogram and the factors included are incomplete, this also has led to questions about the accuracy of those nomograms [18, 19]. None of these studies had complete pathological information, such as VI, nerve invasion and lymphatic invasion, although nerve invasion and lymphatic invasion were not an independent risk factor in this study. Our study included not only the baseline information of the patients, but also the postoperative pathological information of the patients such as the VI. This greatly increases the sensitivity and specificity of this model. This nomogram can help identify high-risk groups for synchronous LM after surgery and help clinicians conduct targeted screening and individualized treatment.

According to previous studies, the prevalence of CRC LM is more common than other metastases, such as the brain or bone [20, 21] and the prevalence of LM was more than 20% [22, 23]. However, the prevalence in this study was less than 20% which was consistent with one study conducted by Manfredi and his colleagues in France [24]. In this study, the incidence of elderly patients was significantly lower than that of young patients, with an incidence of only 13.2%. Because in the past few years, as colonoscopy screening has become more widespread, more and more early signs of cancer have been detected, leading to higher rates of disease in younger people and reducing the prevalence of LM. Tumor size has been shown to be an important factor in the development of LM from CRC [11], while little effect of tumor size was found in this study which might be partly attributed to sample size. Therefore, factors contributing to the development of LM after CRC should be identified and a screening method developed to determine the risk of LM in patients with postoperative CRC.

This study showed that tumor site, T stage, N stage, VI, preoperative CEA level, and CA-199 level were significantly associated with LM development, which were seldom reported before. In our nomogram, colon cancer (CC) is found to be more likely to metastasize than rectal cancer. The most common mechanism is that the metastatic pattern is different. In CC, most mesenteric drainage enters the hepatic portal vein system and the rectal venous-collected blood flows into the systemic circulation [25]. And right-sited CC is found to be more likely to metastasize than left-sited CC, which is consistent with previous articles [11, 23, 24], it was reported that the discrepancy was caused by molecular biological differences [26, 27]. In addition, it is well known that high T stage, N stage, and tumor marker levels represent the malignancy of the tumor, indicating a high likelihood of LM. To some extent, it may explain the difference in prognosis, but this complex issue needs further research.

Since VI is closely related to progression and recurrence of the disease, both the Association of Directors of Anatomic and Surgical Pathology and the College of American Pathologists emphasis is placed on documenting VI during routine pathological examination of cancer specimens. It is reported that the pathologic detection rate of VI was 23% [28]. The proliferation of tumor cells requires energy and nutrition, tumor and other cells secrete VEGF, angiopoietin-like protein, and corresponding inflammatory cells to promote the formation of new blood vessels, thus providing an important channel for these functions [29]. Invasion of blood vessels by tumor cells is a key step in LM. *Fujii T* and *Xie W* et al. found that VI is closely related to the depth of tumor invasion and degree of differentiation [30, 31]. A previous study has shown that VI is significantly associated with metastasis and high recurrence rates [32]. Furthermore, VI has been shown to be associated with reduced overall survival (OS) and disease-free survival (DFS), serving as a strong prognostic indicator [33]. These studies indicate that VI is an essential factor in LM of CRC. The inclusion of this factor improves the accuracy and persuasiveness of our model.

In addition, our research has certain research significance and advantages. Our model mainly identified patients at high risk of developing LM after surgery. For example, a right-sited (40 points) CC patient with VI (32 points), T4 stage (100 points), N2 (62 points), CEA level is positive (58 points), and CA199 level is negative (0 points) has a total of 292 points (high-risk group), resulting the diagnostic possibility is 0.38. In high-risk groups, where there may be micrometastases that were not detected before surgery, the enhanced computed tomography and monitoring should be justified after surgery. In addition, for high-risk patients, positron emission tomography computed tomography (PET-CT) and invasive procedures such as needle biopsy are also recommended for definite diagnosis if there is an undefined low-density liver lesion, because they are expensive and are not justified for general screening. If a positive liver result is found in a targeted examination, we can change the treatment plan in time so as not to delay the disease such as radiofrequency ablation and surgery [34–36]. Of course, this study only provides a reference rather than a guide, and specific decisions should be made based on clinical practice.

The study has several limitations. First of all, since this study is a retrospective study, involving 3190 patients from 2012 to 2020, there may be a possibility of inaccuracy due to the small amount of data, and inevitably there is observer and confusion bias. The current results require further validation in prospective clinical studies. Second, although our model has internal validation, there is a lack of external validation to further determine the model’s accuracy. Third, some underlying factors such as gene status are unknown [37]. The inclusion of these important factors can further improve the effectiveness of the nomogram.

In conclusion, this study performed prediction of synchronous LM in patients undergoing CRC surgery. Therefore, if patients with preoperative diagnosis of colorectal cancer, no LM and underwent surgery are identified as a high-risk patient by nomogram, we should enhance post-operative imaging of the liver, such as enhanced computed tomography, MRI and positron emission tomography computed tomography (PET-CT). It can help clinicians timely detect the disease progression of patients and take effective interventions to improve the quality of life of patients. This is crucial.

## Data Availability

The study data of validation cohort used and/or analyzed during the current study are available from the Second Affiliated Hospital of Harbin Medical University, China.
